# Dose reduction of docetaxel avoids the usage of pegfilgrastim in docetaxel plus ramucirumab therapy for recurrent nonsmall cell lung cancer

**DOI:** 10.1002/cnr2.1793

**Published:** 2023-02-02

**Authors:** Kosuke Hamai, Shinya Miyaka, Shinpei Tada, Suguru Fujita, Tetsu Hirakawa, Mirai Matsumura, Sayaka Ueno, Takuya Tanimoto, Nobuhisa Ishikawa

**Affiliations:** ^1^ Department of Respiratory Medicine Hiroshima Prefectural Hospital Hiroshima Japan; ^2^ Department of Respiratory Medicine Hiroshima General Hospital Hiroshima Japan; ^3^ Department of Molecular and Internal Medicine Hiroshima University Hiroshima Japan

**Keywords:** docetaxel, febrile neutropenia, pegfilgrastim, ramucirumab

## Abstract

**Background:**

Pegfilgrastim is recommended in docetaxel plus ramucirumab (DTX + RAM) therapy for recurrent nonsmall cell lung cancer (NSCLC) because of the associated frequency of febrile neutropenia (FN). However, the FN occurs less frequently when the dose of DTX is reduced because of other adverse events, such as appetite loss and oral mucositis.

**Methods and Results:**

Twenty‐two patients with recurrent NSCLC who received DTX + RAM therapy at the Hiroshima Prefectural Hospital. The cut‐off value which is the most unlikely to cause FN without the combined use of pegfilgrastim was set using a receiver operating characteristic (ROC) curve. This was created according to the dose of DTX and the presence or absence of the onset of FN. We compared the incidence of FN when a DTX dose above and below the cut‐off value was used. The ROC curve showed that 48 mg/m^2^ was the best cut‐off value that predicted whether FN was likely to occur when pegfilgrastim was not used concurrently. The incidence of FN was 26.1% for DTX ≥48 mg/m^2^ and 5.1% for DTX <48 mg/m^2^.

**Conclusions:**

Pegfilgrastim can be discontinued when the dose of DTX is reduced to <48 mg/m^2^ due to nonhematological toxicities.

## 1 INTRODUCTION

Docetaxel plus ramucirumab (DTX + RAM) therapy is a standard treatment for recurrent nonsmall cell lung cancer (NSCLC). In the REVEL trial, DTX + RAM therapy had a median progression free survival (PFS) of 4.5 months and a median overall survival (OS) of 10.5 months, which was significantly prolonged compared with DTX alone (*p* < .0001, *p* = .023, respectively). However, DTX + RAM therapy tends to be more toxic.^1^ The most notable toxicity of DTX + RAM therapy is febrile neutropenia (FN). The incidence of FN was 16% in the REVEL study, and 34.2% in a phase II study in Japan.^2^ Thus, the use of pegfilgrastim is recommended for regimens with an FN incidence of 20% or higher.^3^ In addition to FN, DTX + RAM therapy has many adverse effects, such as loss of appetite and oral mucositis, which may require dose reduction or discontinuation of the drug. The incidence of FN in DTX + RAM therapy when the drug dosage is reduced is unknown, moreover, it is not known whether the use of pegfilgrastim can be avoided with a reduction in the DTX dose. Therefore, based on the data of lung cancer patients who received DTX + RAM therapy at the Hiroshima Prefectural Hospital, we retrospectively investigated the dose of DTX that does not cause FN without the use of pegfilgrastim.

## 2 METHODS

Twenty‐two patients with recurrent NSCLC who received DTX + RAM therapy at the Hiroshima Prefectural Hospital were included in this study. The maximum dose at which FN did not occur without pegfilgrastim was detected retrospectively for every patient. DTX doses less than that maximum dose were not adopted for a receiver operating characteristic (ROC) curve analysis because it was expected that FN would not occur with these lower dose (Table S1). In each treatment cycles using a dose of DTX above the maximum dose, the ROC curve, which showed whether FN occurred or not, was created. The cut‐off value that is the most unlikely to cause FN without the combined use of pegfilgrastim was set using this ROC curve. We compared the incidence of FN when the DTX dose was higher and lower than the cut‐off value. The study protocol was approved by the Institutional Review Board and the Ethics Committee of the Hiroshima Prefectural Hospital.

## 3 RESULTS

**FIGURE 1:**
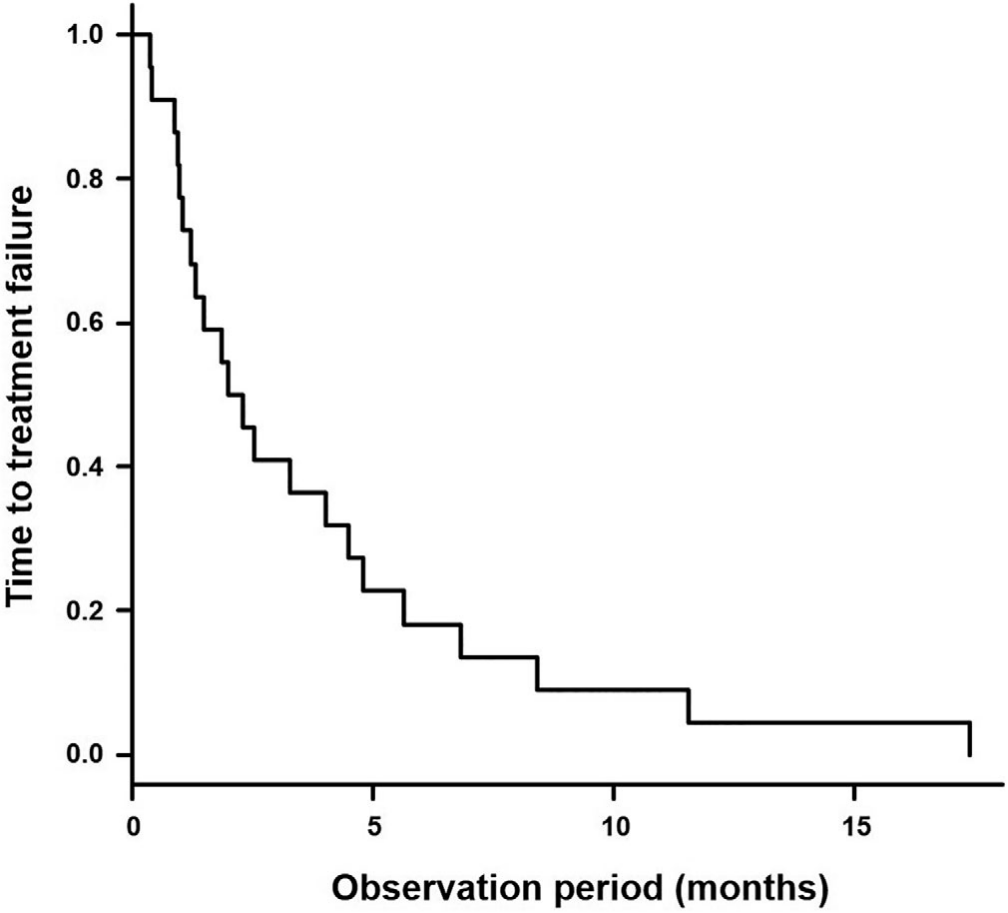
A Kaplan–Meier curve showing the time‐to‐treatment‐failure (TTF) in the 22 enrolled patients.

A total of 89 cycles of DTX + RAM therapy were administered to 22 patients with advanced recurrent lung cancer. The patient characteristics are shown in Table 1. The age of the patients was 63.5 ± 11.3 (mean ± SD) years, and 19 of the 22 patients were male. The histological subtypes were adenocarcinoma in 12 cases, squamous cell carcinoma in six cases, pleomorphic cancer in three cases, and undifferentiated cancer in 1 case. Ten patients received DTX + RAM therapy as a second‐line treatment, 10 as a third‐line treatment, and two as a fourth‐line treatment. They received 1–19 cycles of chemotherapy (median 3 cycles). Of these 89 cycles of chemotherapy, 27 used pegfilgrastim and 62 did not. Thirteen patients discontinued DTX + RAM therapy due to adverse events, and nine patients due to progressive disease or death from lung cancer. The median time‐to‐treatment‐failure was 2.2 months (95% confidence interval (CI): 1.0–4.6 months) (Figure 1). The relevant simmer plot is shown in Figure S1.

**TABLE 1:** Patient characteristics

	(*n* = 22)
Age, mean ± SD (median, range)	63.5 ± 11.3 (66.5, 42–82)
Sex, M/F	19/3
Performance status, 0/1/2	7/12/3
Nonsmoker/smoker	3/19
Histology, Ad/Sq/others	12/6/4
Treatment line, 2nd/3rd/4th~	10/10/2
Treatment cycles, median (range)	3 (1–19)

**FIGURE 2:**
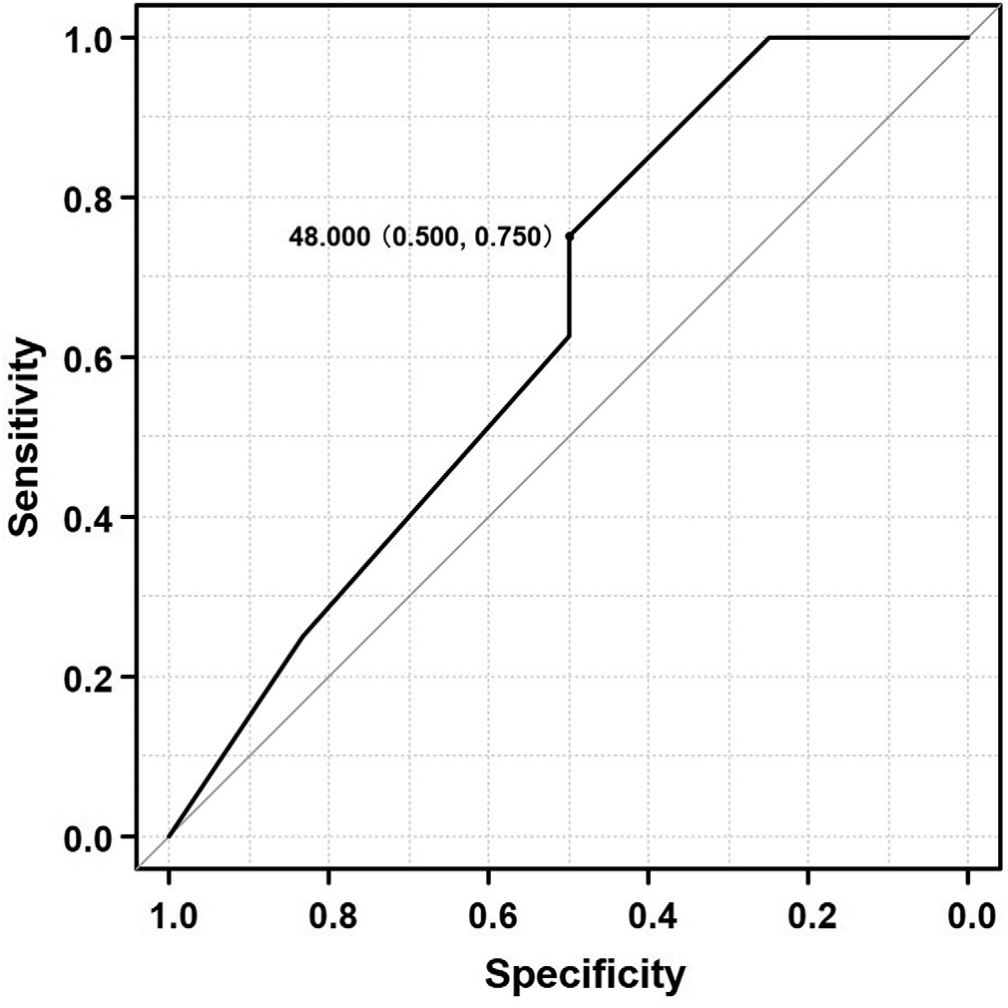
The ROC curve was plotted with 62 cycles of DTX + RAM therapy without pegfilgrastim. The analysis showed that 48 mg/m^2^ was the best cut‐off dose that determined whether FN was likely to occur when pegfilgrastim was not used concurrently. The area under the ROC curve was 0.673 (95% CI: 0.435–0.911), the sensitivity was 50%, and the specificity was 85.7%.

The ROC curve created according to the dose of DTX, and the presence or absence of the onset of FN showed that 48 mg/m^2^ was the best cut‐off value that determined whether FN was likely to occur or not when pegfilgrastim was not used (Figure 2). The area under the ROC curve was 0.635 (95% CI: 0.395–0.876), the sensitivity was 50%, and the specificity was 75%. When pegfilgrastim was not used, the incidence of FN was 26.1% for DTX ≥48 mg/m^2^ and 5.1% for DTX <48 mg/m^2^. The incidence of FN when DTX was used in combination with pegfilgrastim was 3.7%.

## 4 DISCUSSION

FN is a serious and sometimes fatal adverse event related to chemotherapy treatment. ASCO guidelines recommend the use of pegfilgrastim when administering chemotherapy regimens associated with an FN incidence of ≥20%.^3^ DTX + RAM therapy is the standard treatment for advanced recurrent NSCLC. The use of pegfilgrastim is recommended when administering DTX + RAM therapy, because the FN incidence was reported to be 16% in the REVEL study^1^ and 34.2% in a phase II trial in Japan.^2^ However, although FN can be prevented by using pegfilgrastim, other adverse effects such as loss of appetite and oral mucositis may remain problematic, and it may eventually be necessary to reduce the dose of DTX. The REVEL trial included criteria for DTX dose reduction in response to adverse events. In East Asia, enrolled patients began with a DTX dose of 60 mg/m^2^, and those who experienced FN, severe cutaneous reaction, or Grade 3 or 4 nonhematological toxicities had the DTX treatment withheld until toxicity was resolved, following which DTX treatment was resumed at 50 mg/m^2^ for the remainder of the study. Although a DTX dose <50 mg/m^2^ was not allowed in the REVEL trial, in clinical practice, if both the toxicity and antitumor effect persist after single dose reduction, further dose reduction of the cytotoxic agent and treatment continuation may be selected. In such a situation, a lower DTX dose may not cause FN.

This study showed that a dose reduction of DTX to less than 48 mg/m^2^ may enable the discontinuation of pegfilgrastim. In this study, “sensitivity,” which showed the probability of developing FN at DTX dose ≥48 mg/m^2^, was 50%. In fact, with DTX dose ≥48 mg/m^2^, the probability of developing FN was reduced to 26.1%. Therefore, the use of pegfilgrastim was recommended only for the DTX dose ≥48 mg/m^2^ setting. On the contrary, “1‐specificity,” which showed the probability of developing FN at DTX <48 mg/m^2^, was 14.3%. Actually, the probability of developing FN at DTX <48 mg/m^2^ was reduced to 5.1%. Thus, when the DTX dose is reduced to 48 mg/m^2^ or less due to toxicity, DTX + RAM therapy can be safely administered without using pegfilgrastim.

It is not the intention of this study to recommend DTX dose reduction simply for avoiding the use of pegfilgrastim. Reckless DTX dose reduction may prevent the maintenance of adequate antitumor efficacy. Moreover, Kasahara et al.^4^ demonstrated that the use of pegfilgrastim reduced the incidence of FN to 5%, with a median PFS of 6.6 months and a median OS of 18.4 months. Therefore, the use of pegfilgrastim should ideally be continued during standard DTX + RAM therapy. However, pegfilgrastim is a very expensive drug, therefore, if the DTX dose has to be reduced due to grade 3–4 nonhematological toxicity, and especially when the DTX dose is reduced below the level specified in past studies, considering discontinuation of pegfilgrastim may be necessary from a cost‐effectiveness standpoint.

This study had several limitations. It was a retrospective analysis conducted in a single facility. Next, the area under the ROC curve (AUC) used in this analysis was less than 0.7, probably because of the small sample size. An AUC between 0.70 and 0.80 is typically considered “acceptable,” whereas a value exceeding 0.80 is typically considered “excellent.”^5^ Thus, reliability remains debatable. In addition, setting a cut‐off value of “48 mg/m^2^” may not necessarily be eligible or valid, because, apart from DTX dosage, patient characteristics, prior therapy, and baseline white blood cell counts were not considered. However, our retrospective analysis suggests that pegfilgrastim discontinuation when the dose of DTX is reduced due to nonhematological toxicity contributes to the maintenance of the medical economy, and may reduce the burden of hospital visits for patients. However, even when pegfilgrastim can be discontinued when the dose of DTX is reduced due to nonhematological toxicity, careful follow‐up is warranted even in such situations.

## AUTHOR CONTRIBUTIONS

**Kosuke Hamai:** Conceptualization (lead); data curation (lead); formal analysis (lead); investigation (equal); methodology (lead); project administration (lead); resources (lead); software (lead); validation (lead); visualization (lead); writing – original draft (lead); writing – review and editing (lead). **Shinya Miyake:** Investigation (supporting). **Shinpei Tada:** Investigation (supporting). **Suguru Fujita:** Investigation (supporting). **Tetsu Hirakawa:** Investigation (supporting). **Mirai Matsumura:** Investigation (supporting). **Sayaka Ueno:** Investigation (supporting). **Takuya Tanimoto:** Investigation (supporting). **Nobuhisa Ishikawa:** Conceptualization (supporting); investigation (supporting); project administration (supporting); supervision (lead); writing – review and editing (supporting).

## CONFLICT OF INTEREST STATEMENT

The authors report no conflicts of interest in connection with this article.

## ETHICAL STATEMENT

This observational study was performed in accordance with the Declaration of Helsinki. The Ethics Committee of Hiroshima Prefectural Hospital approved the study protocol (approval date: January 27, 2022; approval number: R3‐28‐8).

## Supporting information


**TABLE S1.** The association between the DTX dose and occurrence of FN in each patient. The doses of DTX inducing FN without pegfilgrastim were adopted for ROC curve analysis.

**FIGURE S1.** Swimmer plots of the 22 enrolled patients that received DTX + RAM therapy.

## Data Availability

Data sharing is not applicable to this article as no new data were created or analyzed in this study.
