# Supplementary material for: Dose reduction of docetaxel avoids the usage of pegfilgrastim in docetaxel plus ramucirumab therapy for recurrent nonsmall cell lung cancer
Source: Cancer Rep (Hoboken). 2023 Feb 2;6(4):e1793. doi: 10.1002/cnr2.1793 (PMC10075288; doi:10.1002/cnr2.1793)
Supplement: Supplementary file 1 — TABLE S1. The association between the DTX dose and occurrence of FN in each patient. The doses of DTX inducing FN without pegfilgrastim were adopted for ROC curve analysis. [file CNR2-6-e1793-s001.docx]

**Supplementary Table.S1.** The association between the DTX dose and occurrence of FN in each patient. The doses of DTX inducing FN without pegfilgrastim were adopted for ROC curve analysis.

| Patient No. | DTX (mg/m^2^) | pegfilgrastim | FN | cycles | adopted for ROC curve analysis |
| --- | --- | --- | --- | --- | --- |
| 1 | 60 | not used | occurred | 1 | adopted |
|  | 50 | not used | occurred | 1 | adopted |
| 2 | 60 | not used | occurred | 1 | adopted |
| 3 | 48 | used | not occurred | 2 | not adopted |
|  | 42 | not used | not occurred | 2 | adopted |
| 4 | 50 | not used | not occurred | 1 | adopted |
| 5 | 50 | used | not occurred | 1 | not adopted |
|  | 45 | not used | occurred / not occurred | 1 / 9 | adopted / adopted |
| 6 | 50 | not used | occurred | 1 | adopted |
|  | 45 | used | not occurred | 1 | not adopted |
|  | 45 | not used | not occurred | 4 | adopted |
|  | 40 | not used | not occurred | 5 | not adopted |
| 7 | 50 | not used | not occurred | 9 | adopted |
|  | 48 | not used | not occurred | 10 | not adopted |
| 8 | 50 | not used | occurred | 1 | adopted |
| 9 | 45 | not used | occurred | 1 | adopted |
| 10 | 50 | not used | not occurred | 2 | adopted |
| 11 | 50 | not used | not occurred | 3 | adopted |
| 12 | 60 | not used | not occurred | 1 | adopted |
|  | 60 | used | not occurred | 1 | not adopted |
| 13 | 60 | not used | not occurred | 1 | adopted |
|  | 50 | not used | not occurred | 1 | not adopted |
|  | 45 | not used | not occurred | 1 | not adopted |
| 14 | 40 | not used | not occurred | 1 | adopted |
| 15 | 60 | used | not occurred | 1 | not adopted |
|  | 50 | used | not occurred | 1 | not adopted |
|  | 40 | used | not occurred | 2 | not adopted |
|  | 40 | not used | not occurred | 3 | adopted |
| 16 | 45 | used | not occurred | 2 | not adopted |
|  | 40 | used | not occurred | 1 | not adopted |
| 17 | 48 | not used | occurred | 1 | adopted |
| 18 | 60 | used | not occurred | 2 | not adopted |
|  | 54 | used | not occurred | 1 | not adopted |
| 19 | 54 | used | not occurred | 1 | not adopted |
| 20 | 60 | used | not occurred | 3 | not adopted |
|  | 50 | used | not occurred | 2 | not adopted |
|  | 45 | not used | not occurred | 1 | adopted |
| 21 | 60 | used | occurred / not occurred | 1 / 2 | not adopted |
| 22 | 48 | used | not occurred | 3 | not adopted |

**Supplementary Figure S1.** Swimmer plots of the 22 enrolled patients that received DTX + RAM therapy.
