# Supplementary figures and images for: Dose reduction of docetaxel avoids the usage of pegfilgrastim in docetaxel plus ramucirumab therapy for recurrent nonsmall cell lung cancer
Source: Cancer Rep (Hoboken). 2023 Feb 2;6(4):e1793. doi: 10.1002/cnr2.1793 (PMC10075288; doi:10.1002/cnr2.1793)

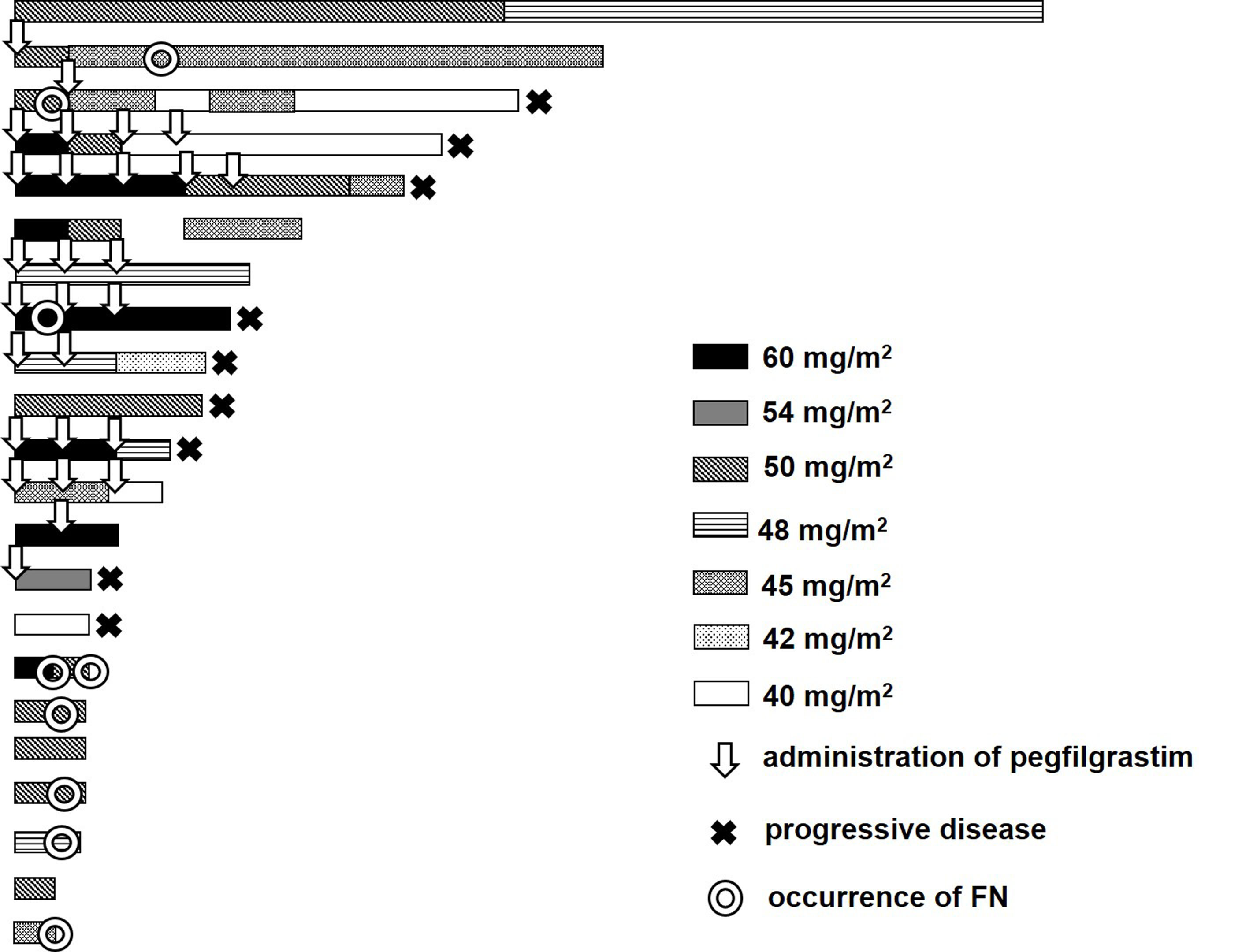

Supplement: Supplementary file 2 — FIGURE S1. Swimmer plots of the 22 enrolled patients that received DTX + RAM therapy. [file CNR2-6-e1793-s002.jpg]
